# A Fluorescent Protein Scaffold for Presenting Structurally Constrained Peptides Provides an Effective Screening System to Identify High Affinity Target-Binding Peptides

**DOI:** 10.1371/journal.pone.0103397

**Published:** 2014-08-01

**Authors:** Tetsuya Kadonosono, Etsuri Yabe, Tadaomi Furuta, Akihiro Yamano, Takuya Tsubaki, Takuya Sekine, Takahiro Kuchimaru, Minoru Sakurai, Shinae Kizaka-Kondoh

**Affiliations:** 1 Graduate School of Bioscience and Biotechnology, Tokyo Institute of Technology, Yokohama City, Japan; 2 Center for Biological Resources and Informatics, Tokyo Institute of Technology, Yokohama City, Japan; University of Essex, United Kingdom

## Abstract

Peptides that have high affinity for target molecules on the surface of cancer cells are crucial for the development of targeted cancer therapies. However, unstructured peptides often fail to bind their target molecules with high affinity. To efficiently identify high-affinity target-binding peptides, we have constructed a fluorescent protein scaffold, designated gFPS, in which structurally constrained peptides are integrated at residues K131–L137 of superfolder green fluorescent protein. Molecular dynamics simulation supported the suitability of this site for presentation of exogenous peptides with a constrained structure. gFPS can present 4 to 12 exogenous amino acids without a loss of fluorescence. When gFPSs presenting human epidermal growth factor receptor type 2 (HER2)-targeting peptides were added to the culture medium of HER2-expressing cells, we could easily identify the peptides with high HER2-affinity and -specificity based on gFPS fluorescence. In addition, gFPS could be expressed on the yeast cell surface and applied for a high-throughput screening. These results demonstrate that gFPS has the potential to serve as a powerful tool to improve screening of structurally constrained peptides that have a high target affinity, and suggest that it could expedite the one-step identification of clinically applicable cancer cell-binding peptides.

## Introduction

Tumor-specific peptides, which can bind to molecules on the surface of cancer cells with high affinity, endow anti-cancer agents (including small chemical drugs and radio-labeled imaging agents) with target specificity and contribute to active drug targeting [1], [2]. Although several peptides that bind to cancers—including breast cancer, prostate cancer, melanoma, and pancreatic cancer—have been identified [1], [3]–[6], most of the peptides that were selected by *in vitro* screening failed to bind to cancer cells *in vivo* [1]. Thus, an easy and reliable screening system that identifies highly specific target-binding peptides that would be applicable *in vivo* is needed.

Peptide-display techniques are commonly used to screen target-binding peptides, primarily because they can evaluate diverse peptide libraries more quickly and with more ease than methods that use chemically synthesized and purified libraries. In peptide-display techniques, peptide libraries are fused with a cell surface protein and displayed on the surface of phages, bacteria, or yeast [7]–[9]. Although these techniques have a high quantitative processing capacity and have facilitated the identification of many candidate peptides, most of the selected peptides were not functional *in vivo*. One possible explanation for this discrepancy is that the displayed peptides are not structurally constrained. The low structural flexibility of constrained peptides reduces the entropic cost of target binding, forcing them to adopt a unique conformation that endows them with higher affinity and specificity for a target than unstructured peptides [10], [11]. Thus, the use of a suitable molecular scaffold that provides peptides structural constraints could greatly improve the screening efficiency of peptide-display techniques for target-specific peptides.

Several protein scaffolds have been reported, including Kunitz domain-containing proteins [12], cytotoxic T lymphocyte-associated antigen 4 [13], the stefin A triple mutant [14], and the Trp cage motif-containing proteins [15]. These scaffolds have been produced by genetic fusion of scaffold protein genes with the peptide encoding sequences, resulting in conformational integration of these peptides following protein expression. Such scaffolds have been used to successfully identify novel target-binding peptides *in vitro* [12]–[15]. However, conformational fluctuation of the incorporated peptides has not been evaluated, and it is unclear whether these scaffolds can be suitable for the presentation of a constrained peptide.

Green fluorescent proteins (GFPs) have also been used as protein scaffolds; their intrinsic fluorescence allows real-time, quantitative monitoring of binding and, therefore, enables easy evaluation of the binding affinity of each peptide. *Aequorea victoria* GFP, enhanced GFP (EGFP), and superfolder GFP (sfGFP) [16] have been examined as scaffolds for peptide presentation. GFPs have a highly rigid structure [17], and their fluorescence is maintained after peptide integration into several loop structures [18]–[22], suggesting that a wide range of peptides could be introduced into GFP molecules without loss of fluorescence. However, when a protease-recognition sequence was integrated into a loop of EGFP, the mutant proteins were completely cleaved by proteases [23], indicating that the backbone of integrated peptides had the structural freedom to allow access to proteases. To date, no protein scaffold has been established for the presentation of structurally constrained peptide libraries.

Here, we describe a fluorescent protein scaffold—gFPS—that provides integrated peptides with a constrained structure enabling easy screening for target-binding peptides with high affinity and specificity. gFPS was constructed from sfGFP, which has a much higher folding capacity than GFP [16]. To determine a suitable site in sfGFP for the presentation of structurally constrained peptides, molecular dynamics (MD) simulations and proteolysis assays were performed on sfGFP mutants using an integrated caspase-3 recognition peptide. High-level resistance to proteolysis implies high structural constraint, because peptides cannot easily enter the narrow substrate-binding pocket of proteases when the backbone structure is rigid [24]. The endo-type protease caspase-3 is a suitable protease for this purpose because it has a narrow and deep substrate-binding pocket, and substrate peptides must fit into the pocket for access to the catalytic center [24]–[26]. After these analyses, gFPS was defined as a sfGFP-based scaffold that can present a structurally constrained peptide that is integrated into the sfGFP K131–L137 site. By using gFPSs that presented established breast cancer-targeting peptides, we evaluated the specific binding ability of the peptides based on fluorescence intensity. Finally, to probe the applicability of gFPS in conventional screening systems, we applied gFPS to yeast-display technology and confirmed that gFPS was displayed on the cell surface of yeast cells, and is therefore applicable for efficient screening of target-specific peptides.

## Results

### Construction of a fluorescent protein scaffold, gFPS

**Figure 1:**
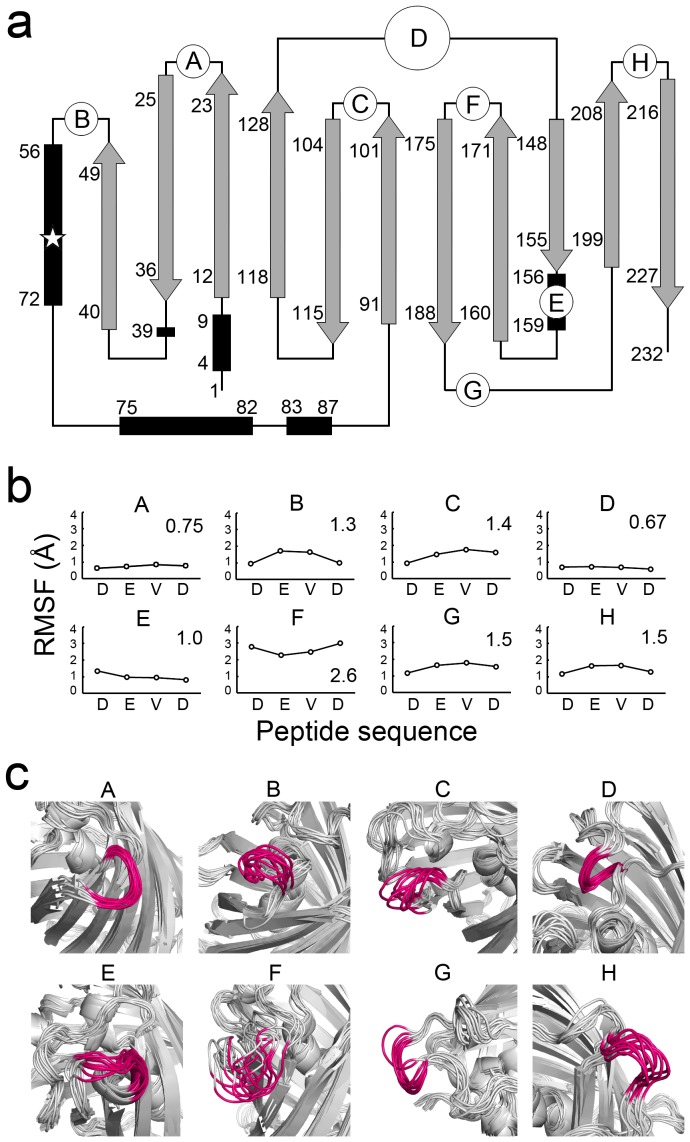
Fluctuation of integrated peptides. (a) A topology diagram of sfGFP. β-strands, α-helices, and the chromophore are represented by gray arrows, black rectangles, and a white star, respectively. The DEVD peptide was integrated into the A (D23/G24), B (G51/K52), C (D102/D103), D (G134–L137), E (Q157/K158), F (D173/G174), G (G189/D190), and H (E213/K214) sites. (b) Root mean square fluctuation (RMSF) of the integrated peptides. The RMSF values represent the atomic fluctuations of each residue throughout 4.5–9.0 ns trajectories. Average fluctuation distances are also indicated. (c) Superimposed structures at every 0.5 ns throughout the 4.5–9.0 ns trajectory for mutant proteins in MD simulations. The integrated peptides are highlighted in magenta.

Bright fluorescence is a powerful tool for the objective detection of substances in screening libraries. To establish a peptide screening system for structurally constrained peptides, we used sfGFP to create a GFP-based scaffold, gFPS, which efficiently restricts peptide fluctuation without a loss of fluorescence following peptide integration. One method used to confirm the structural constraint imposed on the peptide backbone is the examination of proteolytic resistance of the integrated peptide. We first integrated a caspase-3 recognition peptide, Asp-Glu-Val-Asp (DEVD) [27], into sfGFP at eight different sites *in silico* (A–H in Fig. 1a) and calculated the structural fluctuation of the peptides. The root mean square deviations (RMSDs) were gradually increased in the initial phase, and subsequently reached a plateau after 4.5 ns in all the systems (Fig. S1a); thereafter, 4.5–9.0 ns trajectories in MD simulations were used for structural evaluation. The β-can structures of sfGFP were maintained (Fig. S1b), and the backbone fluctuation among the DEVD-integrated sfGFP mutants was <3 Å, except in the C-terminal region (Fig. S1c). The peptide integration sites at A, D, and E showed low fluctuation (average fluctuations: A = 0.75 Å, D = 0.67 Å, E = 1.0 Å) (Fig. 1b), and each loop of these proteins adopted a specific structure (Fig. 1c). By contrast, the peptide integration sites at B, C, G, and H showed slightly larger fluctuations (1.3–1.5 Å) and the peptide integration site at F showed the largest fluctuation (2.6 Å) with many structural variations (Figs. 1b and 1c). These results indicate that the DEVD fluctuation varied depending on the integration site, and that integration at site D (residues 134–137) imposed the highest degree of structural constraint on the peptide. Thus, site D was considered to be the most suitable integration site.

**Figure 2:**
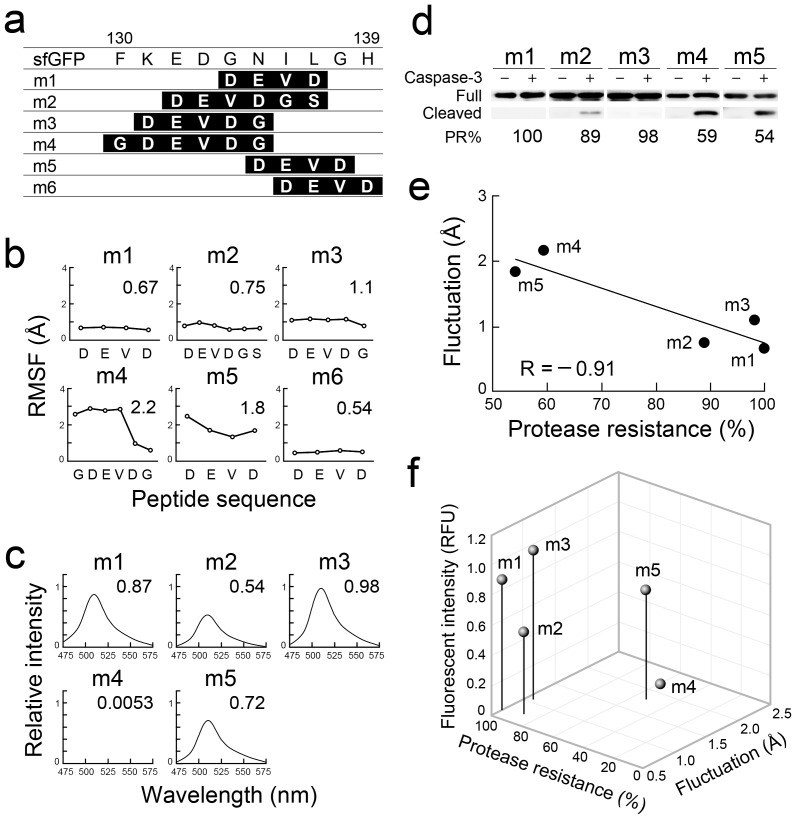
Analysis of sfGFP mutants with peptide integration around site D. (a) Peptide sequences of the sfGFP and mutants. The integration region of each mutant is highlighted in black and the integrated peptide sequences are shown in white letters. (b) Root mean square fluctuation (RMSF) values of peptides sequences of m1–m6 are shown. Average fluctuation distances are also indicated. (c) Fluorescence spectra obtained following excitation at 480 nm (m1–m5). The fluorescence intensity of sfGFP at 510 nm was used as a reference. Relative intensities of mutant proteins are also indicated. (d) Proteolytic resistance of mutants m1–m5. Cleaved fragments were analyzed after 24-h treatment with caspase-3 using western blotting. Protease resistance (PR%) was calculated from the band intensity of cleaved fragments (Cleaved) compared with that of remaining full-length proteins (Full). (e) Linear correlation between the average fluctuation of the integrated peptides and protease resistance. (f) Comparison of sfGFP mutant tolerance based on the position of integration. The relative fluorescent intensity (RFU) at 510 nm compared with that of sfGFP, the protease resistance (%) evaluated using western blotting, and structural fluctuation (Å) of the various peptides calculated by MD simulation are shown.

Next, we determined the optimal position of peptide integration around site D for the construction of a functional protein scaffold. We constructed mutants m1–m6, which are sfGFP mutants integrated with DEVD-containing peptides at various positions around site D (residues 130–139) (Fig. 2a). Note that m1 is the same mutant as “D” indicated in Figs. 1b and 1c, and S1. MD simulations examining m2–m6 revealed that the peptides integrated in m2, m3, and m6 were structurally constrained, whereas the m4 and m5 peptides showed fluctuation (Figs. 2b and S2a–d). We next examined the fluorescence intensity and resistance to proteolysis; m1, m2, and m3 were strongly fluorescent and showed high resistance to proteolysis, whereas both florescence and the resistance to proteolysis were not observed for m4, while m5 showed an increased susceptibility to proteolysis (Figs. 2c and 2d), and m6 could not be purified because it was insoluble (Fig. S3). In m1–m5, the average fluctuation of the integrated peptides (Fig. 2b) and protease resistance (Fig. 2d) showed a high negative correlation (R = −0.91; Fig. 2e). This supported the idea that fluctuation of the integrated peptides could be evaluated based on protease sensitivity. Moreover, all the protease-resistant mutants (m1, m2, and m3) showed bright fluorescence (Fig. 2f). These results indicate that peptides integrated into the m1–m3 peptide integration sites, which correspond to residues 131–137, would be structurally constrained. The sfGFP scaffold presenting exogenous peptides at this site (residues 131–137) is hereafter termed gFPS.

**Figure 3:**
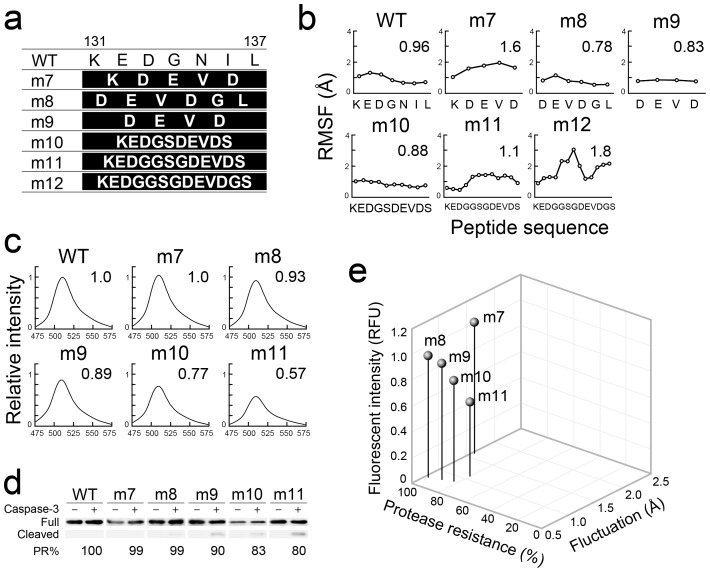
Analysis of gFPSs containing polypeptides of various lengths at K131–L137. (a) Peptide sequences of gFPSs. The amino acid sequence of sfGFP is shown at the top. The integrated peptide sequences of m7–m12 are shown in white letters. (b) Root mean square fluctuation (RMSF) values of K131–L137 in sfGFP and m7–m12 are shown. Average fluctuation distances are also indicated. (c) Fluorescence spectra following excitation at 480 nm for sfGFP and m7–m11. The fluorescence intensity of sfGFP at 510 nm was used as a reference. Relative intensities of gFPSs are also indicated. (d) Proteolytic resistance of sfGFP and m7–m11. Cleaved fragments were analyzed after 24-h treatment with caspase-3 by western blotting. Protease resistance (PR%) was calculated from the band intensity of cleaved fragments (Cleaved) compared with that of remaining full-length proteins (Full). (e) Comparison of gFPS tolerance with regard to the peptide length. The relative fluorescent intensity (RFU) at 510 nm compared with that of sfGFP, the protease resistance (%) evaluated using western blotting, and structural fluctuation (Å) of the various peptides calculated by MD simulation are shown.

For further evaluation of gFPS efficacy as a scaffold for large-sized libraries, the permissible range of peptide length that could be presented in gFPS was evaluated using m7–m12 (Fig. 3a). To determine the lower limit of peptide length, gFPSs presenting 5, 6, and 4 amino acids (aa) were constructed and designated m7, m8, and m9, respectively (Fig. 3a). The integrated peptides in these scaffolds were structurally constrained (Fig. 3b), these mutants were strongly fluorescent (Fig. 3c), and the peptides retained anti-proteolytic properties (Figs. 3d and 3e), indicating that libraries wherein the peptides integrated in gFPS are more than four aa in length can be screened efficiently. The upper limit of peptide length that could be presented in gFPS was investigated using m10, m11, and m12, in which peptides that were 10, 12, and 13 aa in length were integrated, respectively (Fig. 3a). Analysis of the fluctuation of integrated peptides, and the fluorescence and protease resistance of the mutant proteins revealed that the integrated peptides in m10 and m11 are structurally constrained (Figs. 3b, 3c, 3d, and 3e). However, based on *in silico* analysis, conformation of integrated peptide in m12 was found to have highly variable (Figs. S2d) and m12 could not be purified because the protein became insoluble (Fig. S3), indicating that the upper limit of peptide length that can be integrated into gFPS is 12 aa. These results demonstrated that gFPS can be used to present structurally constrained peptides ranging from 4 to 12 aa in length.

### Evaluation of tumor-binding peptides in gFPS

**Figure 4:**
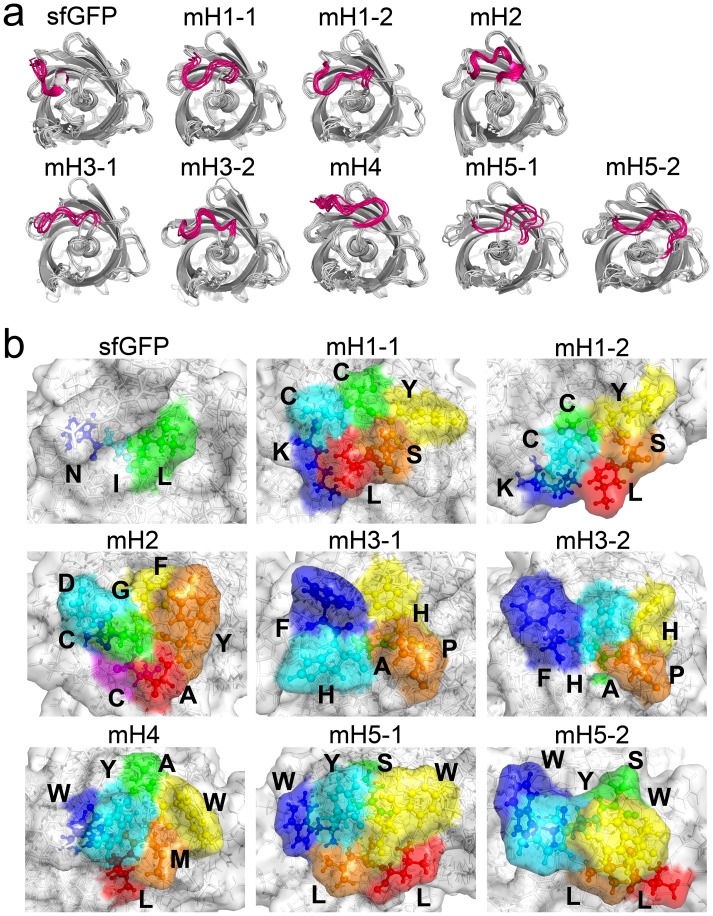
Analysis of the gFPSs containing HER2-BPs. (a) Superimposed structures of sfGFP, mH1, mH2, mH3, mH4, and mH5 at every 0.5 ns throughout the 4.5–9.0 ns trajectory of the MD simulations. The K131–L137 integration sites are highlighted in magenta. (b) Representative surface structures for sfGFP, mH1, mH2, mH3, mH4, and mH5. Amino acids (N135–L137) in the sfGFP and integrated HER2-BPs of mH1–mH5 are also shown using the ball and stick model. These peptides are highlighted in blue, cyan, green, yellow, orange, red, and magenta for the 1st–7th amino acids, respectively.

To verify the utility of gFPS as a scaffold for presenting structurally constrained peptides in screens for target-specific peptides, we tested peptides that were previously demonstrated to bind to human epidermal growth factor receptor type 2 (HER2), a breast cancer marker. The HER2-binding peptides KCCYSL, CDGFYAC, FHAHP, WYAWML, and WYSWLL [28]–[30]—designated as HER2-BP 1, 2, 3, 4, and 5, respectively—were presented in gFPSs, which were designated mH1, mH2, mH3, mH4, and mH5, respectively. The 4.5–9.0 ns MD simulation trajectories revealed that the structure and backbone fluctuations of these proteins were similar to those of m1 (Fig. S4). Snapshot analysis showed that HER2-BPs in mH1, mH3, and mH5 took on two constrained structures; fluctuations of both structures were small (Fig. 4a), suggesting that they had two stable constrained structures. HER2-BPs in mH1, mH4, and mH5 were of the same aa length but showed differing conformations (Fig. 4a), indicating that integrated constrained peptides in gFPS took on unique structures depending on their aa sequence. In addition, almost all side chains of the HER2-BPs appeared on molecular surface of gFPSs, except for the C residue of mH2 and the A residue of mH3 (Fig. 4b), indicating that these peptides could interact with target molecules even in the constrained form.

**Figure 5:**
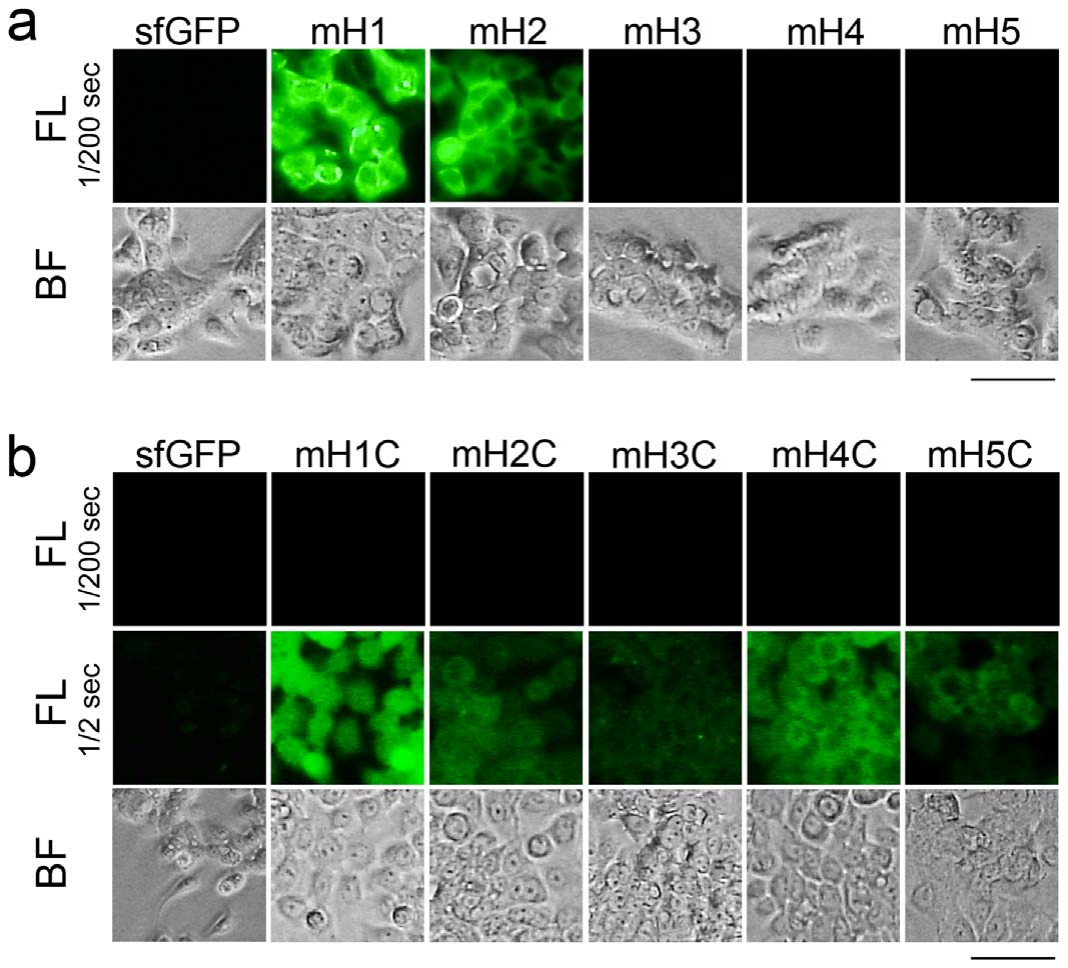
Binding assays for the gFPSs containing HER2-BPs. (a) Fluorescence (FL) and bright field (BF) micrographs of HER2-positive N87 cells treated with sfGFP, mH1, mH2, mH3, mH4, and mH5 for 16 h. Exposure time  = 1/200 s. Bar  = 50 µm. (b) FL and BF micrographs of HER2-positive N87 cells treated with sfGFP, mH1C, mH2C, mH3C, mH4C, and mH5C for 16 h. Exposure time  = 1/200 s or 1/2 s. Bar  = 50 µm.

The binding affinity of these gFPSs was evaluated using HER2-positive N87 and HER2-negative HeLa cells. All the gFPSs retained fluorescence (Fig. S5a), confirming the previous results. When mH1 and mH2 were added to cultured N87 and HeLa cells, strong fluorescence was observed for the N87 cells but not for the HeLa cells (Figs. 5a and S5b), indicating that HER2-BP 1 and 2 integrated into gFPS retained their ability to bind to the cell surface HER2 protein. By contrast, mH3, mH4, and mH5 showed almost no binding to N87 cells (Figs. 5a and S5b). These results correlate strongly with those of *in vivo* studies; HER2-BP 1 and 2 have been used as the HER2-targeting modules of imaging probes and as drugs in mouse models [28], [31]–[33]. *In vivo* studies of HER2-BP 3, 4, and 5 have not yet been reported. To evaluate the importance of structural constraint, HER2-BPs 1–5 were fused to the C-terminal of sfGFP and the resultant mutants were designated mH1C–mH5C, respectively. All the mutant proteins exhibited bright fluorescence (Fig. S5a) and bound to N87 cells (Figs. 5b and S6), consistent with previous studies [28]–[30]. However, their affinity to HER2-positive N87 cells was very weak, and a low level of binding to HER2-negative HeLa cells was also observed (Fig. S6), indicating that loss of structural constraint in peptides reduces their target specificity. These results confirm that the structural constraint of peptides ensures high specificity and affinity to their targets.

### Evaluation of gFPS presented on the cell surface of yeast

**Figure 6:**
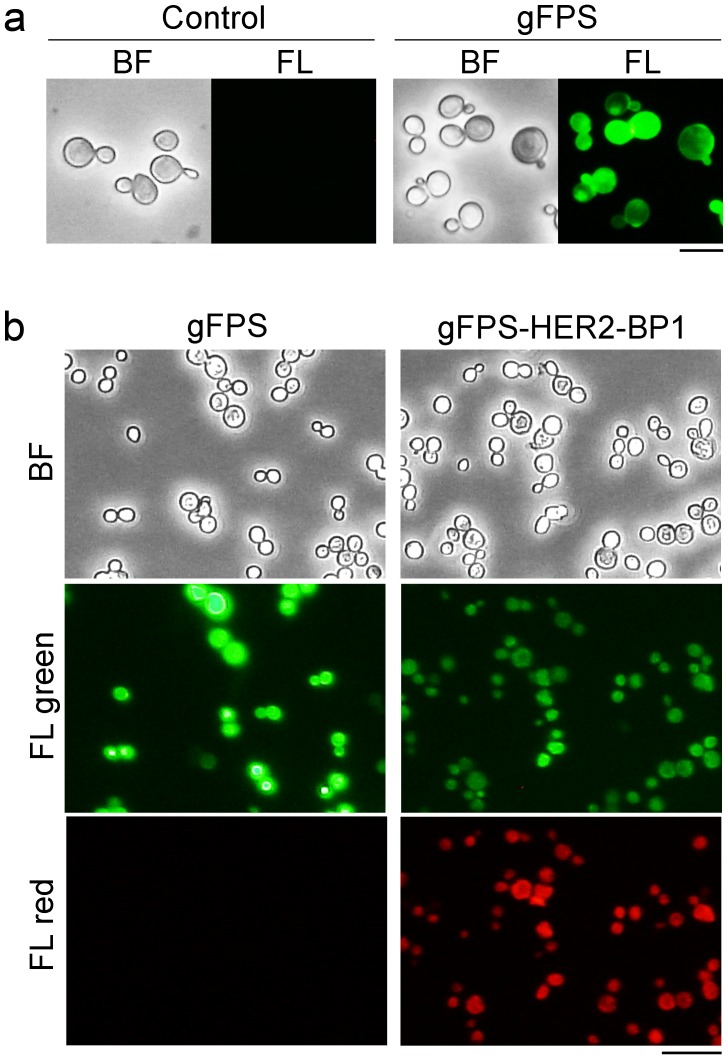
Molecular display of gFPS on the cell surface of yeast. (a) Bright field (BF) and fluorescence (FL) micrographs of yeast cells harboring empty control (pULD1) or gFPS-displaying plasmids (pULD1-gFPS). Bar  = 10 µm (b) BF, green fluorescence (FL green), and red fluorescence (FL red) micrographs of gFPS- or gFPS-HER2-BP 1-displaying yeast cells treated with R-HER2-ECD for 3 h. Bar  = 20 µm.

To examine potential applications of gFPS in a conventional high-throughput screening system, gFPS molecules were displayed on the cell surface of yeast. Strong fluorescence was observed in cells harboring the plasmid encoding gFPS, but not in the cells harboring the control plasmid (Fig. 6a), indicating that gFPS was successfully displayed on the yeast cell surface. Furthermore, when the yeast cells displaying HER2-BP 1 in gFPS (gFPS-HER2-BP 1) were treated with rhodamine-labeled extracellular domain of HER2 protein (R-HER2-ECD), red fluorescence was observed only in the cells displaying gFPS-HER2-BP 1, and not in the cells displaying gFPS alone (Fig. 6b). These results clearly demonstrate that gFPS represents a unique scaffold design for peptide display in a yeast display system and strongly suggest that this scaffold can be used in a wide range of high-throughput screening systems used to identify peptides that have high target affinity and specificity.

## Discussion

**Figure 7:**
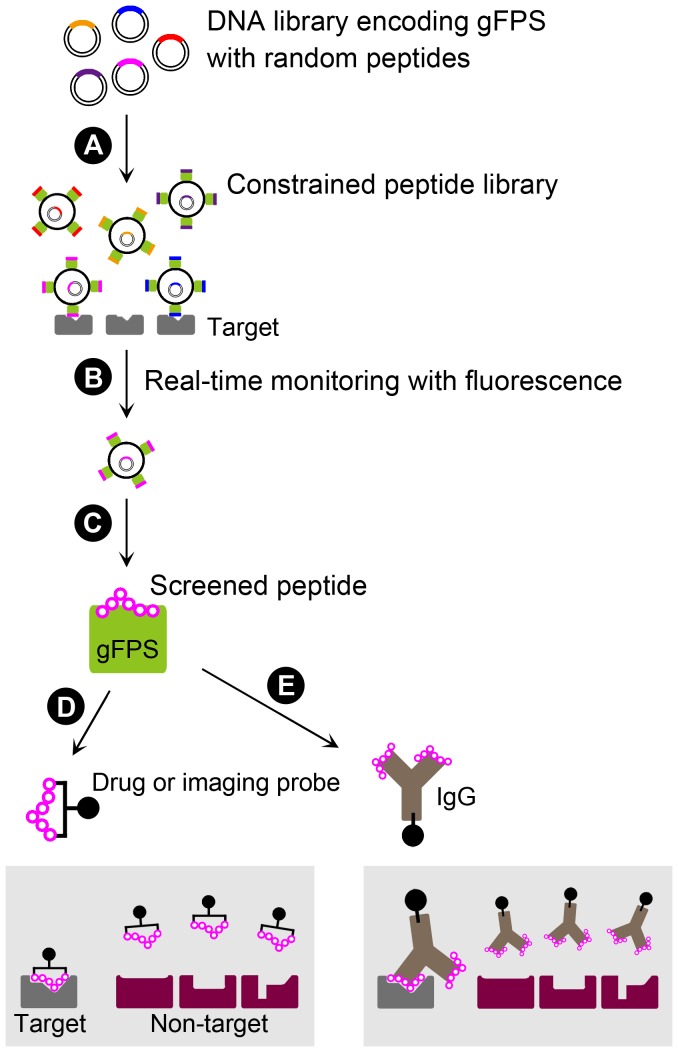
A one-step peptide screening system using gFPS. DNA libraries encoding gFPS with random peptides are introduced into the host (e.g., phage, bacteria, yeast, mammalian cells), resulting in the display of structurally constrained peptides on the host cell surface (A). The host cells that express peptides that bind to the target are easily selected based on gFPS fluorescence (B). The plasmid in the selected host is amplified and the DNA sequence of the corresponding peptide is determined (C). The identified peptides can be tested *in vivo* using structurally constrained forms such as circularized peptides (D) or following integration into *in vivo* scaffolds such as immunoglobulin G (IgG) molecules (E). These peptides retain unique structures that specifically bind to the target molecule with high affinity *in vivo*.

In this report, we suggest the utilization of a novel protein scaffold, gFPS, for the presentation of structurally constrained peptides to effectively identify high affinity target-binding peptides in a conventional high-throughput screening system (Fig. 7).

Tight folding of the GFP polypeptide is required to facilitate chromophore formation and to confer resistance to proteolysis [23], [34], [35]. gFPSs presenting peptides of various lengths remained brightly fluorescent, indicating that their structural rigidity is similar to that of sfGFP; accordingly, the gFPSs also showed strong resistance to proteolysis (Figs. 2f and 3e). An additional mutation in the N-terminal side neighboring site D (131–137)—as demonstrated by m4, which differs from m3 by the additional mutation of F130G—caused loss of both resistance to proteolysis and fluorescence (Fig. 2f). When the F131 residue of superglo GFP (sgGFP), which corresponds to F130 of sfGFP, was substituted with other amino acids, the resultant F131I and F131L mutants retained bright fluorescence, the F131A mutant showed reduced fluorescence, and that of the F131T, F131P, F131H, F131Q, F131D, and F131K mutants was lost [36]. The hydrophobicity of each residue correlates with fluorescence (I [4.5] >L [3.8] >F [2.8] >A [1.8] >G [−0.4] >T [−0.7] >P [−1.6] >H [−3.2] >Q [−3.5]  = D [−3.5] >K [−3.9]) [37], suggesting that hydrophobic interactions between the side chain of F130 of sfGFP (F131 of sgGFP) and surrounding residues are critical for the tight packing of the structure. As a result, m4 lacks the structural rigidity necessary for chromophore formation, and the structural constraint imposed on the integrated peptide is also lost. Two amino acid residues at the C-terminal side of site D (131–137), G138 and H139, may be required to maintain rigid conformation and solubility of sfGFP, respectively, because the m5 mutant (G138→D138; Fig. 2a) lacks resistance to proteolysis (Fig. 2f) and m6 (H139→D139; Fig. 2a) is insoluble (Fig. S3). Collectively, D (131–137) is a unique and suitable site for presentation of structurally constrained peptides without the loss of tight folding.

gFPSs presenting HER2-BP 1 and 2, mH1 and mH2, strongly bind to the HER2-expressing N87 cells, but show no interaction with HER2-negative HeLa cells (Figs. 5a and S5b), indicating that gFPS effectively presents target-binding peptides and can identify high affinity target-binding peptides that may be functional *in vivo*. Note that EGFP cannot be used as a scaffold because the EGFP mutant proteins with these peptides integrated into the site corresponding to site D (131–137) were insoluble (Fig. S7). mH3, mH4, and mH5 exhibited weak affinities toward HER2-expressing cells, probably due to the loss of structural freedom of the peptide in gFPS. Because HER2-BP 3, 4, and 5 are selected from unstructured peptide libraries [3], [30], their binding to HER-2 might be less specific, and based on their structural freedom, they may assume conformations that would result in lax HER2 binding. By contrast, HER2-BP 1 is locally constrained by a disulfide bond, and HER2-BP 2 was originally designed as a cyclic-constrained peptide, indicating that these peptides show maximum target affinity owing to their constrained structures. Taken together, these results demonstrate that it is important to retain structural constraint during the screening process in order to identify high affinity target-binding peptides.

Our findings indicate that gFPS has significant potential as a widely usable peptide presentation platform to improve conventional screening systems. Display technology is one of the most commonly used high-throughput screening systems used to identify target-binding peptides. Because GFPs are already known to be displayed on the cell surface of phages [20], bacteria [8], and yeast [9], gFPS can be used in any of these display systems to develop a constrained peptide library. Moreover, after first screening with a conventional method, quantitative evaluation of binding affinity is used to select peptides *in vitro* using chemically synthesized peptides. However, some peptides cannot be synthesized due to increased hydrophobicity. The gFPS, reported in this study may address this issue; sfGFP was originally introduced as a robustly folded version of GFP that folds well even when fused to poorly folded polypeptides [16], suggesting that various aa sequences can be fused to gFPS without a loss of solubility. These advantages suggest that a screening method using gFPS would be a reliable strategy for the selection of target-binding peptides.

For clinical applications such as active drug targeting tools, the peptides selected by this system must be detached from gFPS because it is immunogenic in itself. There are two possible clinically applicable forms of the detached peptides; the first is a cyclic form that is often used *in vivo* to obtain structural restriction of peptides [10]. As shown in Figure 5a, mH2, which contains a peptide originally designed as a cyclic peptide, shows clear binding activity to HER2-expressing cells, suggesting that cyclic peptides can integrate into gFPS without the loss of binding ability. This suggests that constrained peptides screened using gFPS would be functional in the cyclic form. The second form involves integration into another scaffold that does not elicit an immune response. The hypervariable complementarity-determining region (CDR) of immunoglobulin (Ig) comprises loops between β-sheets with the same architecture as gFPS, and the CDR loops can accept a vast number of aa sequences without a loss of function. Thus, for clinical application of cancer-binding peptides selected using gFPS, Ig molecules might be suitable *in vivo* scaffolds, which allow the peptides to retain their constrained structures (Fig. 7).

In summary, we constructed a highly efficient system for screening peptides with high target affinity using a fluorescent protein scaffold, gFPS. The peptide length applicable in this system that would still allow the retention of fluorescence is 4–12 aa, allowing on-demand construction of various peptide libraries and easy screening of target-specific peptides using fluorescence as an indicator. The results using HER2-targeting peptides demonstrate the potential of gFPS as a presentation scaffold to generate constrained peptide libraries, and suggest that a screening system using gFPS might facilitate identification of clinically applicable target-binding peptides in conventional high-throughput screening systems.

## Materials and Methods

### Human cell lines and culture conditions

Human cervical cancer HeLa cells and human gastric cancer N87 cells were obtained from the RIKEN Bio-Resource Center (Tsukuba, Japan) and ATCC (Manassas, VA, USA), respectively, and maintained at 37°C in 5% FBS Dulbecco's-modified Eagle's medium (Life Technologies, Carlsbad, CA, USA) supplemented with penicillin (100 units/mL) and streptomycin (100 µg/mL) (Nacalai Tesque, Kyoto, Japan).

### Site-directed mutagenesis and expression of sfGFP and its mutants

The complementary DNA (cDNA) encoding sfGFP was prepared by site-directed mutation of EGFP sequence and inserted into the *Bam*HI-*Eco*RI site of pGEX-6P-3 plasmid (GE Healthcare Life Sciences, Buckinghamshire, UK). The cDNA encoding m1–m12, mH1–mH5, or mH1C–mH5C were constructed by site-directed mutation of sfGFP sequence. These plasmids were introduced into BL21-CodonPlus (DE3) cells (Agilent Technologies, Santa Clara, CA, USA) for the protein expression. The sequences of oligo DNA used for the experiments were listed (Table S1).

### Purification of proteins

Fusion proteins were expressed in BL21-CodonPlus (DE3) cells as GST-tagged proteins and purified as previously described [38]. The final proteins were equilibrated in 9.5 mM phosphate buffer, containing 137 mM NaCl and 2.7 mM KCl, pH 7.4 (PBS).

### Proteolysis by caspase-3

The 10 µM sfGFP, m1–m5, and m7–m11 were cleaved by 14 mU/mL human caspase-3 [EC 3. 4. 22. 56] (Sigma-Aldrich, St. Louis, MO, USA) for 24 h at 37°C in the reaction solutions, 20 mM Pipes (pH 7.2) containing 100 mM NaCl, 0.1% CHAPS, 10% sucrose, and 10 mM DTT.

### Measurement of fluorescence

Fluorescence (475 nm–575 nm excited at 480 nm) of 10 µM sfGFP, m1–m5, and m7–m11 in 20 mM Pipes (pH 7.2) containing 100 mM NaCl, 0.1% CHAPS, and 10% sucrose was measured by using GeneQuant 1300 (GE Healthcare Life Sciences) and F-2700 Fluorescence spectrophotometer (Hitachi High-Technologies, Tokyo, Japan), respectively. Fluorescence intensity of 25 µM sfGFP, mH1–mH5, and mH1C–mH5C in PBS was measured using Infinite F500 (Tecan, Männedorf, Switzerland) in 96 well plates with the specific filters (Ex/Em  = 485 nm/535 nm).

### Western blotting

The protein samples were electrophoresed on 12.5% SDS-polyacrylamide gel and transferred to Hybond ECL membranes (GE Healthcare Life Sciences). sfGFP, m1–m5, and m7–m11 were detected with rabbit polyclonal anti-GFP antibody (Cell Signaling Technology, Danvers, MA, USA). The primary antibodies were then reacted with goat monoclonal anti-rabbit IgG antibody, horseradish peroxidase-conjugate (Cell Signaling Technology).

### Molecular dynamics (MD) simulation

The initial coordinates of sfGFP were taken from Protein Data Bank (PDB) structure 2B3P, and the structures of mutant sfGFPs were generated by integrating each peptides into sfGFP using Discovery studio 3.1 (Accelrys, San Diego, CA, USA). Chromophore residues (65–67) of proteins were substituted to GGG sequence for each system. The systems were optimized via energy minimization, and equilibrated with backbone restraints. Then 9.0 ns production runs were performed for trajectory analysis. All the MD simulations were performed using Amber 11 program package [39] on TSUBAME (Global Scientific Information and Computing Center, Tokyo Institute of Technology). The Amber ff99SB force field and the GB/SA implicit solvent model were used. Time step for MD simulations was set to 2 fs with the SHAKE algorithm. A nonbonded cutoff of 999.9 Å was used. The temperature was kept constant at 300 K using Berendsen rescaling method.

### Calculation of the root mean square deviation (RMSD) and the root mean square fluctuation (RMSF)

The RMSDs of Cα atoms from initial structures were calculated using 9.0 ns MD trajectories to monitor the overall dynamics of sfGFP, m1–m12, mH1–mH5, and mH1C–mH5C. The RMSF during 4.5–9.0 ns were also measured to investigate the backbone fluctuations in each system. The RMSDs and RMSFs were calculated using the ptraj module in Amber 11.

### Binding assay of gFPSs toward HER2 positive cells

N87 and HeLa cells (2.0×10^5^ cells/well) were seeded on the slide chamber plate, cultured for 16 h, and fixed by the treatment of 4% paraformaldehyde phosphate buffer solution for 10 min at room temperature. Then the cells were washed by PBS and blocked with 3% BSA/TBS-T for 30 min. After the addition of 2 µM of the purified gFPSs to the chambers, the cells were incubated for 16 h at 4°C and washed. The fluorescent micrograph of the cell samples was observed using Biozero BZ-8100 (Keyence, Osaka, Japan) with the filter of Ex/Em  = 470±40 nm/535±50 nm.

### Yeast strain and media

*Saccharomyces cerevisiae* BJ2168 (MATa prb1-1122 prc1-407 pep4-3 ura3-52 leu2 trp1) was provided by the National Bio-Resource Project (NBRP) of the MEXT, Japan and used to construct yeasts displaying gFPS. Yeast transformants were selected on a synthetic dextrose (SDC) solid medium (0.67% yeast nitrogen base without amino acids, 2% glucose, 0.5% casamino acids, 0.004% L-tryptophan, and 1.5% agar), and then, the resultant colonies were cultivated in a liquid SDC medium at 30°C.

### Molecular display on yeast cell surface

The DNA fragment encoding sfGFP was inserted into *Bgl*II/*Xho*I-digested pULD1 plasmid [40] and referred to as pULD1-gFPS. The plasmid for the display of gFPS-HER2-BP 1 was constructed by site-directed mutation of pULD1-gFPS using the primers listed (Table S1) and termed pULD1-gFPS-HER2-BP 1. Yeasts were transformed using the Frozen-EZ Yeast Transformation-II kit (Zymo Research, Irvine, CA, USA). After the introduction of plasmids, the yeast transformants were selected on a uracil-deficient SDC solid medium.

### Preparation of Rhodamine-labeled HER2-ECD

The DNA fragment encoding extracellular domain of HER2 protein (23–652 residues, HER2-ECD) was inserted into pGEX-6P-3 plasmid. The resultant plasmid was introduced into BL21-CodonPlus (DE3) cells. The HER2-ECD protein was expressed, purified by the same method to mutant sfGFPs, and equilibrated in PBS. Labeling reaction was examined in 1.5 mL PBS containing 7 nmol HER2-ECD, 70 nmol NHS-Rhodamine (Life technologies), and 0.25% dimethylformamide (Wako, Osaka, Japan) at room temperature for 1 h. The Rhodamine-labeled HER2-ECD (R-HER2-ECD) was equilibrated in PBS containing 0.01% Brij 35 (Wako).

### Binding assay of displayed gFPSs toward HER2

The yeast transformants (2.0×10^6^ cells) were washed by PBS and resuspended in 250 µL of binding buffer (PBS containing 0.01% BSA and 0.01% Brij35). After the addition of 0.5 µM of R-HER2-ECD, the cells were incubated at room temperature for 3 h and washed by the binding buffer. The fluorescent micrograph of the cell samples was observed using Biozero BZ-8100 with the filters of Ex/Em  = 470±40 nm/535±50 nm for gFPS and 540±25 nm/605±55 nm for Rhodamine.

## Supporting Information

Figure S1 **Analysis of sfGFP mutants with peptide integration around site A–H.** (a) Time course of root mean square deviation (RMSD) throughout 9.0 ns MD simulation in each system. (b) Average structures throughout the 4.5–9.0 ns simulations for each system. The integrated peptides are highlighted in magenta. (c) Root mean square fluctuation (RMSF) values of each residue of the overall protein.
(TIF)

Figure S2 **Analysis of sfGFP mutants with peptide integration.** (a) Time course of root mean square deviation (RMSD) throughout 9.0 ns MD simulation in each system. (b) Average structures throughout the 4.5–9.0 ns simulations for each system. The integrated peptides in m1–m6 and K131-L137 in sfGFP and m7–m12 are highlighted in magenta. (c) Root mean square fluctuation (RMSF) values of each residue of the overall protein. (d) Superimposed structures at every 0.5 ns throughout the 4.5–9.0 ns of the MD simulations. The integrated peptides in m1–m6 and K131-L137 region in sfGFP and m7–m12 are highlighted in magenta.
(TIF)

Figure S3 **SDS-PAGE analysis of m6 and m12. m6 and m12 were expressed in** ***E. coli*** **.** The cells were disrupted by ultrasonication and centrifuged, then the supernatant and cell pellet were analyzed. Arrowheads indicate expressed proteins.
(TIF)

Figure S4 **Analysis of the gFPS containing HER2-BPs.** (a) Time course of root mean square deviation (RMSD) throughout 9.0 ns MD simulation in each system. (b) Average structures throughout the 4.5–9.0 ns simulations for each system. The integrated peptides are highlighted in magenta. (c) Root mean square fluctuation (RMSF) values of each residue of the overall protein. (d) RMSF values of integrated peptides are shown. Average fluctuation distances are also indicated in the graphs.
(TIF)

Figure S5 **Binding assays for the gFPSs containing HER2-BPs.** (a) Five different HER2-BPs (HER2-BP 1, 2, 3, 4, and 5) were integrated into the gFPS (mH1, mH2, mH3, mH4, and mH5) or fused to C-terminal amino acid of sfGFP (mH1C, mH2C, mH3C, mH4C, and mH5C) and their fluorescence brightness was measured (n = 3) and mean of relative fluorescent intensity ± SEM is shown in the graph. (b) Fluorescence (FL) and bright field (BF) micrographs of HER2-positive N87 cells and HER2-negative HeLa cells treatment with sfGFP, mH1, mH2, mH3, mH4, and mH5 for 16 h. Exposure time is 1/200 sec. Bar  = 200 µm.
(TIF)

Figure S6 **Fluorescence and bright field micrographs of HER2-positive N87 cells and HER2-negative HeLa cells treatment with sfGFP, mH1C, mH2C, mH3C, mH4C, and mH5C for 16 h.** Exposure time is 1/200 sec and 1/2 sec. Bar  = 100 µm.
(TIF)

Figure S7 **SDS-PAGE analysis of EGFP containing HER2-biniding peptides.** The EGFP mutants integrating HER2-BP 1, 2, and 3 (E-mH1, E-mH2, and E-mH3) were expressed in *E. coli*. The cells were disrupted by ultrasonication and centrifuged, then the supernatant and cell pellet were analyzed. Arrow heads indicate expressed wild-type and mutant EGFP proteins.
(TIF)

Table S1 **Sequences of oligo DNAs used for site-directed mutagenesis.**
(DOCX)
