# Supplementary figures and images for: A Fluorescent Protein Scaffold for Presenting Structurally Constrained Peptides Provides an Effective Screening System to Identify High Affinity Target-Binding Peptides
Source: PLoS One. 2014 Aug 1;9(8):e103397. doi: 10.1371/journal.pone.0103397 (PMC4118881; doi:10.1371/journal.pone.0103397)

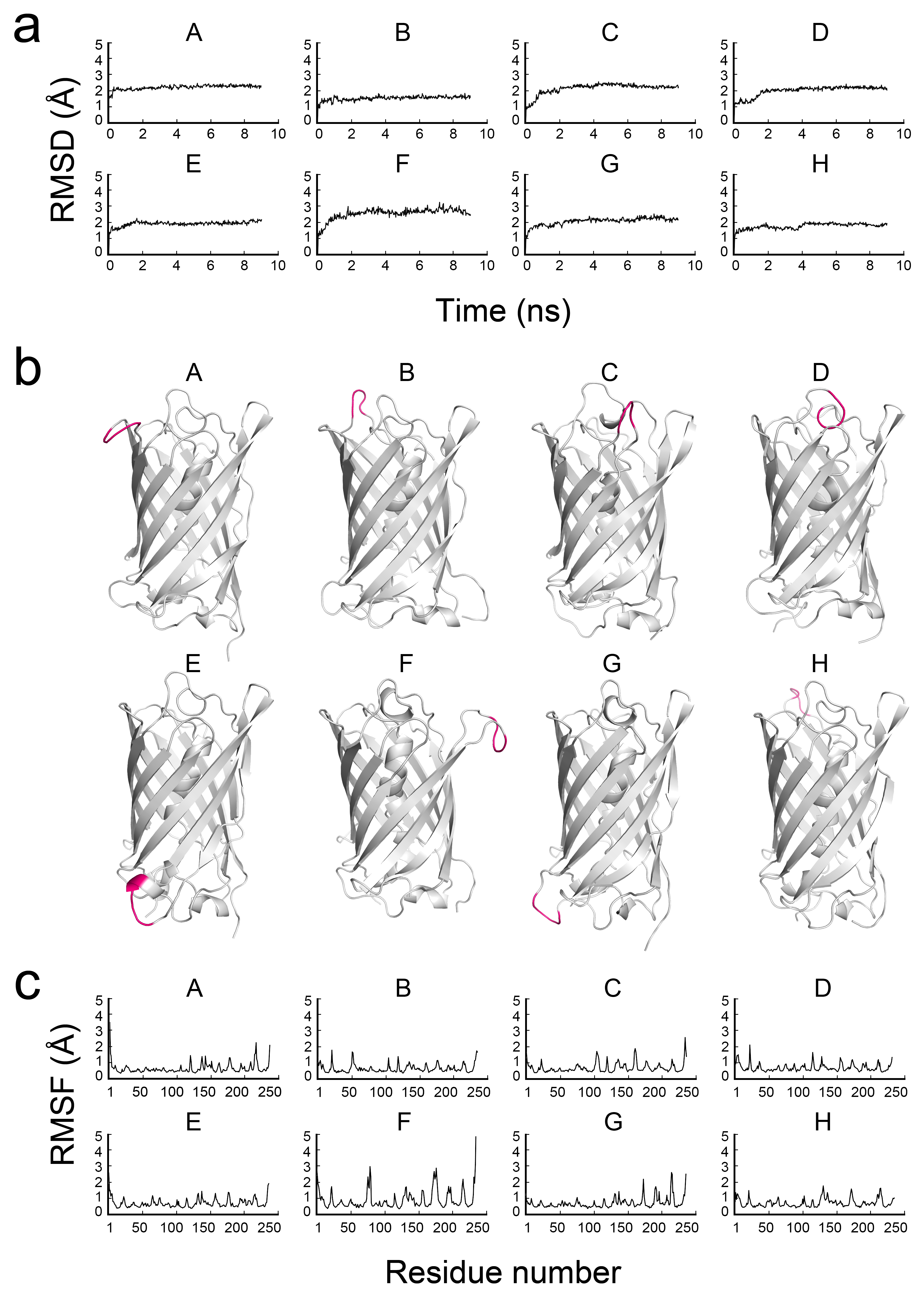

Supplement: Figure S1 — Analysis of sfGFP mutants with peptide integration around site A–H. (a) Time course of root mean square deviation (RMSD) throughout 9.0 ns MD simulation in each system. (b) Average structures throughout the 4.5–9.0 ns simulations for each system. The integrated peptides are highlighted in magenta. (c) Root mean square fluctuation (RMSF) values of each residue of the overall protein. (TIF) [file pone.0103397.s001.tif]

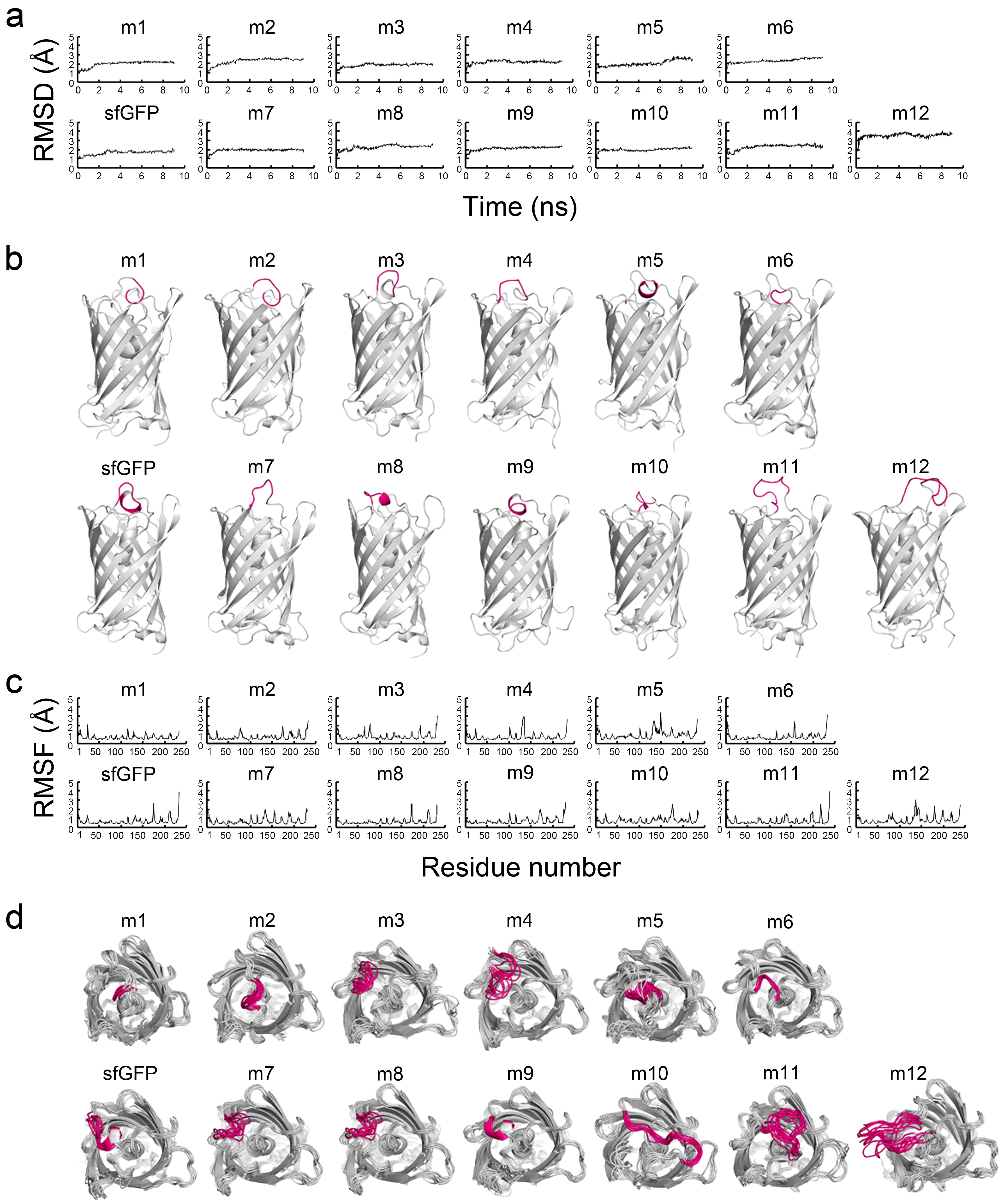

Supplement: Figure S2 — Analysis of sfGFP mutants with peptide integration. (a) Time course of root mean square deviation (RMSD) throughout 9.0 ns MD simulation in each system. (b) Average structures throughout the 4.5–9.0 ns simulations for each system. The integrated peptides in m1–m6 and K131-L137 in sfGFP and m7–m12 are highlighted in magenta. (c) Root mean square fluctuation (RMSF) values of each residue of the overall protein. (d) Superimposed structures at every 0.5 ns throughout the 4.5–9.0 ns of the MD simulations. The integrated peptides in m1–m6 and K131-L137 region in sfGFP and m7–m12 are highlighted in magenta. (TIF) [file pone.0103397.s002.tif]

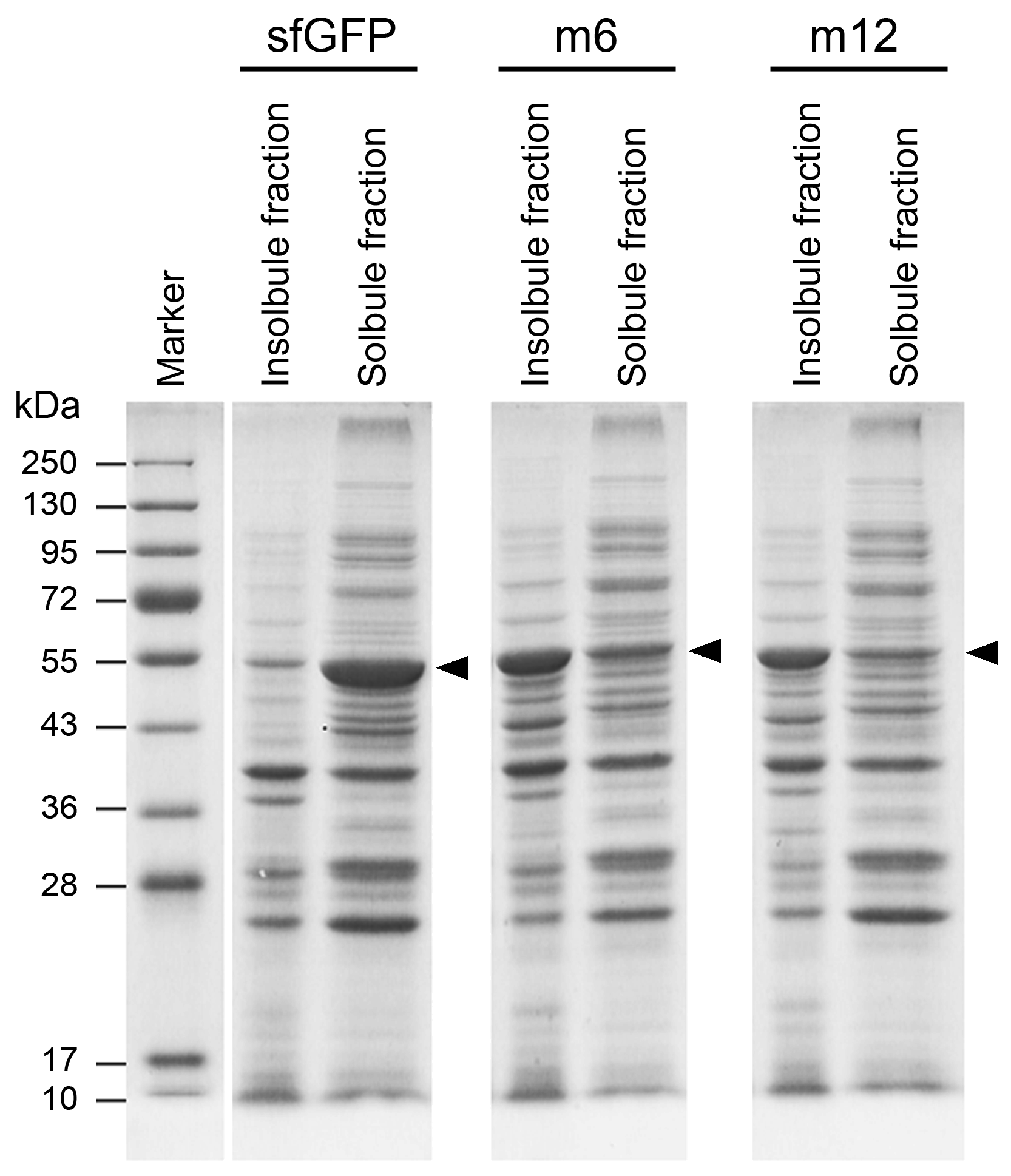

Supplement: Figure S3 — SDS-PAGE analysis of m6 and m12. m6 and m12 were expressed in E. coli . The cells were disrupted by ultrasonication and centrifuged, then the supernatant and cell pellet were analyzed. Arrowheads indicate expressed proteins. (TIF) [file pone.0103397.s003.tif]

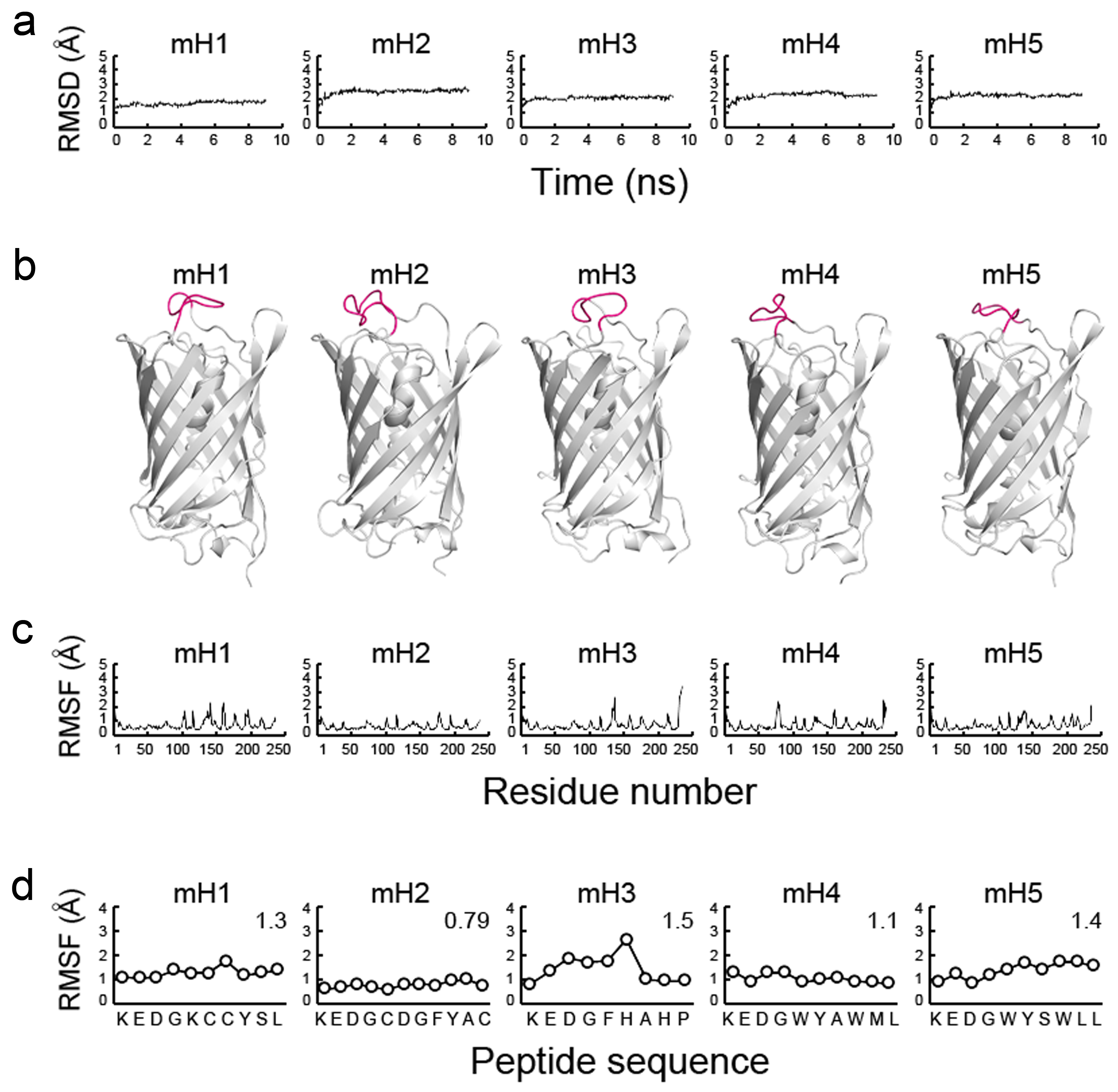

Supplement: Figure S4 — Analysis of the gFPS containing HER2-BPs. (a) Time course of root mean square deviation (RMSD) throughout 9.0 ns MD simulation in each system. (b) Average structures throughout the 4.5–9.0 ns simulations for each system. The integrated peptides are highlighted in magenta. (c) Root mean square fluctuation (RMSF) values of each residue of the overall protein. (d) RMSF values of integrated peptides are shown. Average fluctuation distances are also indicated in the graphs. (TIF) [file pone.0103397.s004.tif]

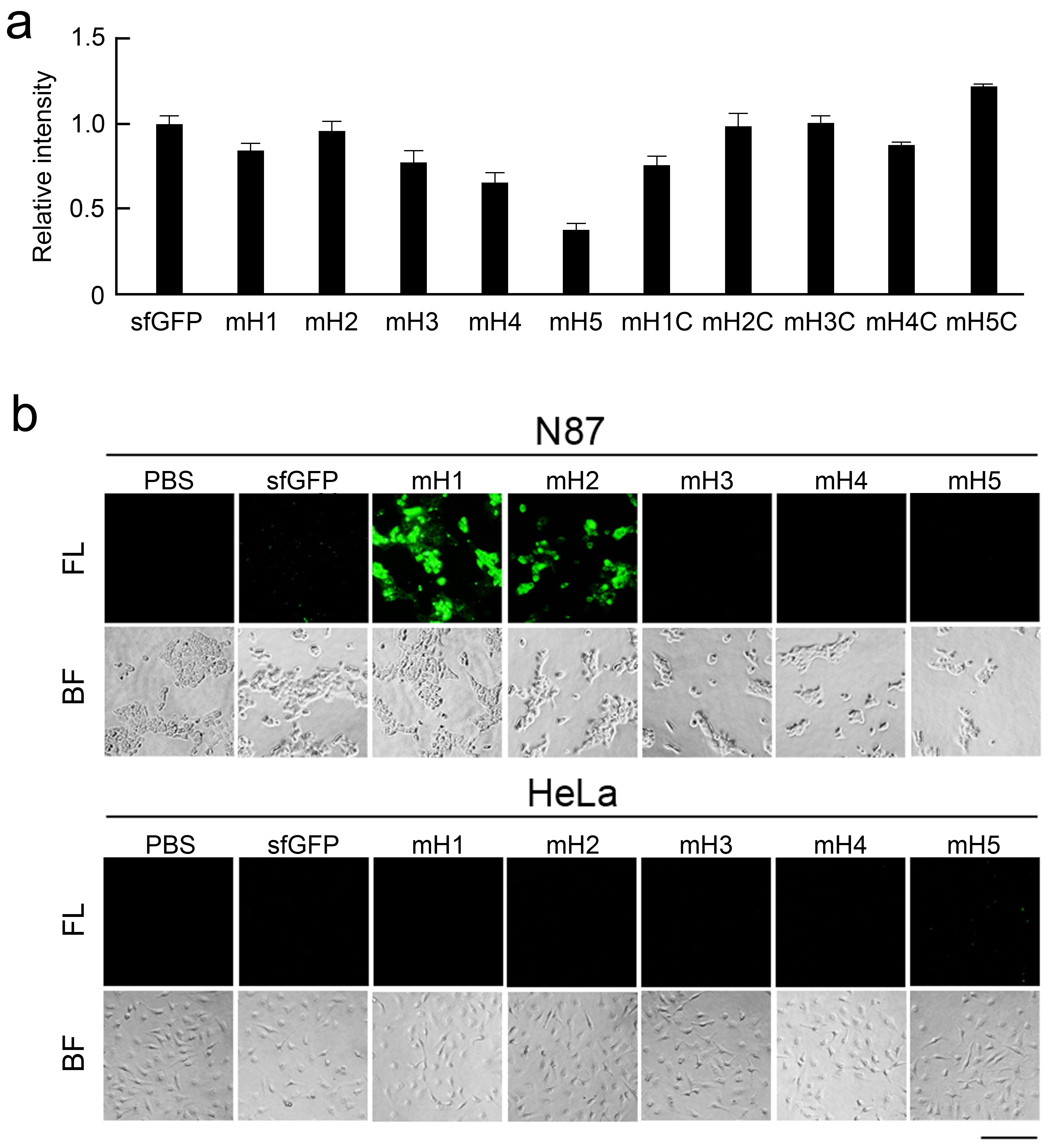

Supplement: Figure S5 — Binding assays for the gFPSs containing HER2-BPs. (a) Five different HER2-BPs (HER2-BP 1, 2, 3, 4, and 5) were integrated into the gFPS (mH1, mH2, mH3, mH4, and mH5) or fused to C-terminal amino acid of sfGFP (mH1C, mH2C, mH3C, mH4C, and mH5C) and their fluorescence brightness was measured (n = 3) and mean of relative fluorescent intensity ± SEM is shown in the graph. (b) Fluorescence (FL) and bright field (BF) micrographs of HER2-positive N87 cells and HER2-negative HeLa cells treatment with sfGFP, mH1, mH2, mH3, mH4, and mH5 for 16 h. Exposure time is 1/200 sec. Bar = 200 µm. (TIF) [file pone.0103397.s005.tif]

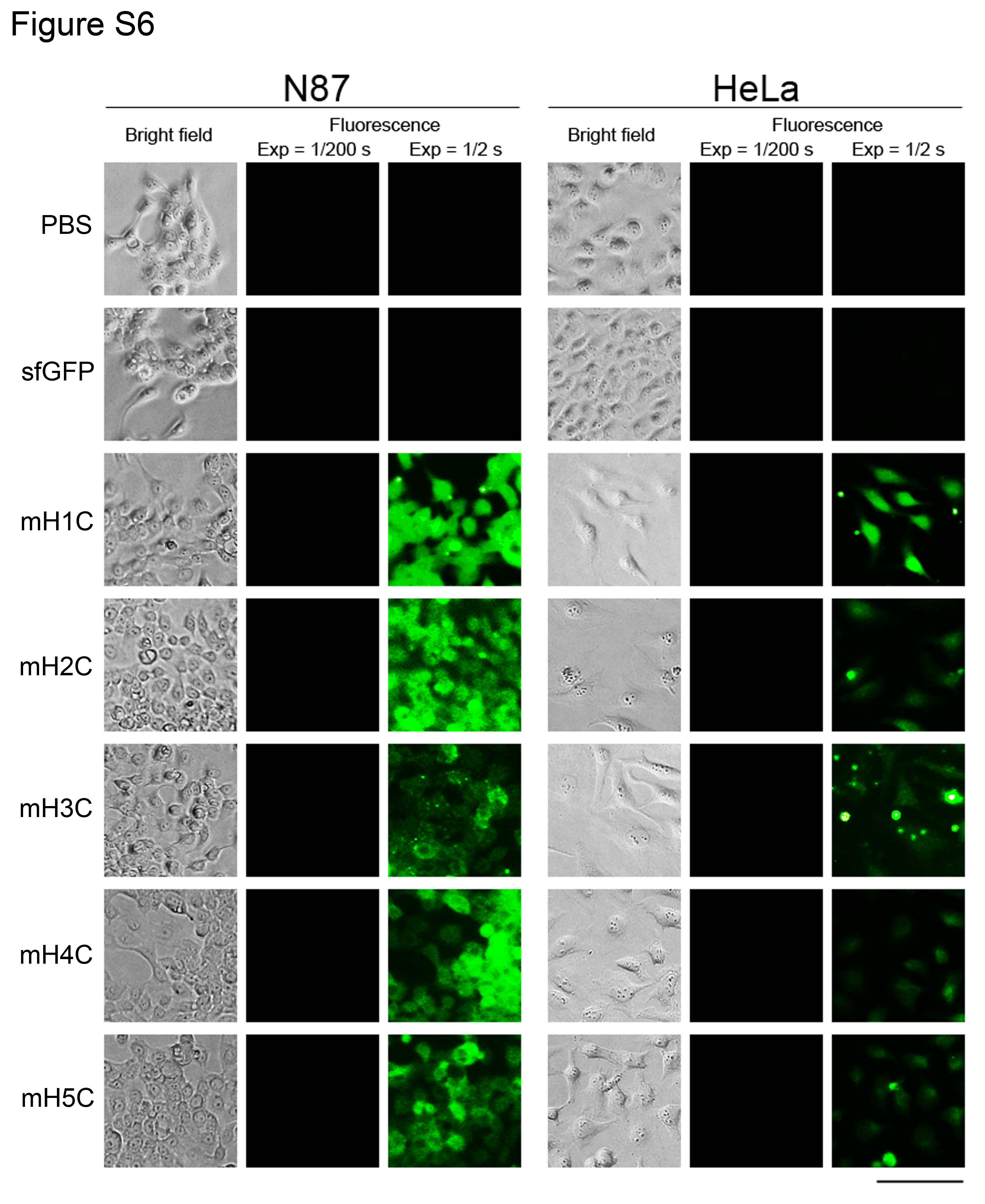

Supplement: Figure S6 — Fluorescence and bright field micrographs of HER2-positive N87 cells and HER2-negative HeLa cells treatment with sfGFP, mH1C, mH2C, mH3C, mH4C, and mH5C for 16 h. Exposure time is 1/200 sec and 1/2 sec. Bar = 100 µm. (TIF) [file pone.0103397.s006.tif]

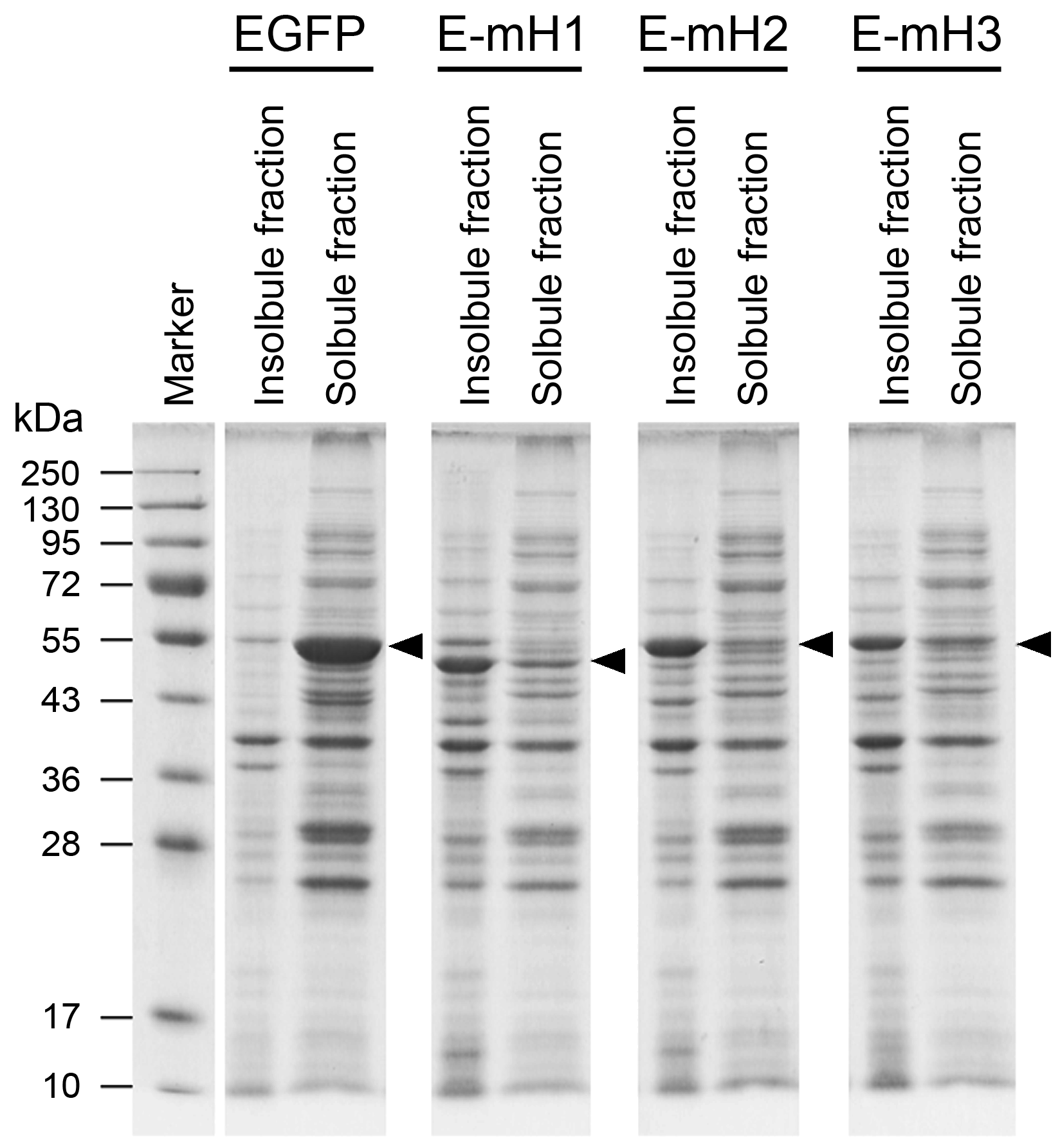

Supplement: Figure S7 — SDS-PAGE analysis of EGFP containing HER2-biniding peptides. The EGFP mutants integrating HER2-BP 1, 2, and 3 (E-mH1, E-mH2, and E-mH3) were expressed in E. coli. The cells were disrupted by ultrasonication and centrifuged, then the supernatant and cell pellet were analyzed. Arrow heads indicate expressed wild-type and mutant EGFP proteins. (TIF) [file pone.0103397.s007.tif]
