# Supplementary material for: A Fluorescent Protein Scaffold for Presenting Structurally Constrained Peptides Provides an Effective Screening System to Identify High Affinity Target-Binding Peptides
Source: PLoS One. 2014 Aug 1;9(8):e103397. doi: 10.1371/journal.pone.0103397 (PMC4118881; doi:10.1371/journal.pone.0103397)
Supplement: Table S1 — Sequences of oligo DNAs used for site-directed mutagenesis. (DOCX) [file pone.0103397.s008.docx]

**Supplementary table**

**Table S1.** Sequences of oligo DNAs used for site-directed mutagenesis

| Mutants | Mutagenesis  (Template cDNA) | Sequence of oligo DNA |
| --- | --- | --- |
| EGFP1 | S30R/Y39N substitution | 5’-CACAAGTTCAGCGTGCGCGGCGAGGGCGAGGGCGATGCCACCAACGGCAAGCTGACCCTG-3’ |
|  | (EGFP) | 5’-CAGGGTCAGCTTGCCGTTGGTGGCATCGCCCTCGCCCTCGCCGCGCACGCTGAACTTGTG-3’ |
| EGFP2 | F99S/N105T substitution | 5’-CAGGAGCGCACCATCAGCTTCAAGGACGACGGCACCTACAAGACCCGCGCC-3’ |
|  | (EGFP1) | 5’-GGCGCGGGTCTTGTAGGTGCCGTCGTCCTTGAAGCTGATGGTGCGCTCCTG-3’ |
| EGFP3 | A206V substitution | 5’-CTGAGCACCCAGTCCGTGCTGAGCAAAGACCCC-3’ |
|  | (EGFP2) | 5’-GGGGTCTTTGCTCAGCACGGACTGGGTGCTCAG-3’ |
| EGFP4 | Y145F substitution | 5’-CAAGCTGGAGTACAACTTCAACAGCCACAACGTC-3’ |
|  | (EGFP3) | 5’-GACGTTGTGGCTGTTGAAGTTGTACTCCAGCTTG-3’ |
| EGFP5 | M153T substitution | 5’-CACAACGTCTATATCACCGCCGACAAGCAGAAG-3’ |
|  | (EGFP4) | 5’-CTTCTGCTTGTCGGCGGTGATATAGACGTTGTG-3’ |
| EGFP6 | V163A substitution | 5’-GAAGAACGGCATCAAGGCGAACTTCAAGATCCGC-3’ |
|  | (EGFP5) | 5’-GCGGATCTTGAAGTTCGCCTTGATGCCGTTCTTC-3’ |
| sfGFP | I171V substitution | 5’-CAAGATCCGCCACAACGTGGAGGACGGCAGCGTG-3’ |
|  | (EGFP6) | 5’-CACGCTGCCGTCCTCCACGTTGTGGCGGATCTTG-3’ |
| m1 | 134-DEVD-137 | 5’-GATGAAGTTGATGAGTACAACTTCAACAGC-3’ |
|  | (sfGFP) | 5’-CAGGATGTTGCCGTCCTC-3’ |
| m2 | 132-DEVDGS-137 | 5’-GATGAAGTTGATGGCAGCGGGCACAAGCTGGAGTAC-3’ |
|  | (sfGFP) | 5’-CTTGAAGTCGATGCCCTT-3’ |
| m3 | 131-DEVDG-135 | 5’-GATGAAGTTGATGGCATC-3’ |
|  | (m4) | 5’-GAAGTCGATGCCCTTCAG-3’ |
| m4 | 130-GDEVDG-135 | 5’-GGCGATGAAGTTGATGGCATCCTGGGGCACAAGCTG-3’ |
|  | (sfGFP) | 5’-GTCGATGCCCTTCAGCTC-3’ |
| m5 | 135-DEVD-137 | 5’-GTTGATCACAAGCTGGAGTAC-3’ |
|  | (sfGFP) | 5’-TTCATCGCCGTCCTCCTTGAA-3’ |
| m6 | 136-DEVD-139 | 5’-GTTGATAAGCTGGAGTACAAC-3’ |
|  | (sfGFP) | 5’-TTCATCGTTGCCGTCCTCCTT-3’ |
| m7 | 132-DEVD-137 | 5’-GATGAAGTTGATGGGCACAAGCTGGAGTAC-3’ |
|  | (sfGFP) | 5’-CTTGAAGTCGATGCCCTT-3’ |
| m8 | 131-DEVDG-136 | 5’-GGCGATGAAGTTGATGGCCTGGGGCACAAGCTG-3’ |
|  | (m4) | 5’-GAAGTCGATGCCCTTCAG-3’ |
| m9 | 131-DEVD-137 | 5’-GATGAAGTTGATGGGCACAAGCTGGAGTAC-3’ |
|  | (m7) | 5’-GAAGTCGATGCCCTTCAG-3’ |
| m10 | 131-KEDGSDEVDS-137 | 5’-TCCGACGAGGTGGACTCCGGGCACAAGCTGGAGTAC-3’ |
|  | (sfGFP) | 5’-GCCGTCCTCCTTGAAGTCGAT-3’ |
| m11 | 131-KEDGGSGDEVDS-137 | 5’-GGGGACGAGGTGGACTCCGGGCACAAGCTG-3’ |
|  | (sfGFP) | 5’-CAGCTTGTGCCCGGAGTCCACCTCGTCCCC-3’ |
| m12 | 131-KEDGGSGDEVDGS-137 | 5’-GGGTCCGGGGACGAGGTGGACGGGTCCGGGCACAAGCTGGAGTAC-3’ |
|  | (sfGFP) | 5’-GCCGTCCTCCTTGAAGTCGAT-3’ |
| mH1 | 135-KCCYSL-137 | 5’-CCGGCGAGTCCGGCGGATCGAGAAGTGGGGCACAAGCTGGAG-3’ |
|  | (sfGFP) | 5’-GCCGTCCTCCTTGAAG-3’ |
| mH2 | 135-CDGFYAC-137 | 5’-CTTCAAGGAGGACGGCTGCGATGGTTTTTATGCGTGCGGGCACAAGCTGGAG-3’ |
|  | (sfGFP) | 5’-CTCCAGCTTGTGCCCGCACGCATAAAAACCATCGCAGCCGTCCTCCTTGAAG-3’ |
| mH3 | 135-FHAHP-137 | 5’-AAATGCTGCTATAGTCTGGGGCACAAGCTGGAG-3’ |
|  | (sfGFP) | 5’-GCCGTCCTCCTTGAAG-3’ |
| mH4 | 135-WYAWML-137 | 5’-TGGTACAGCTGGCTGCTGGGGCACAAGCTGGAGTAC-3’ |
|  | (sfGFP) | 5’-GCCGTCCTCCTTGAAGTC-3’ |
| mH5 | 135-WYSWLL-137 | 5’-TGGTACGCCTGGATGCTGGGGCACAAGCTGGAGTAC-3’ |
|  | (sfGFP) | 5’-GCCGTCCTCCTTGAAGTC-3’ |
| mH1C | 239-GKCCYSL-245 | 5’-GGCAGCCATCATCAC-3’ |
|  | (sfGFP) | 5’-TTACAGACTATAGCAGCATTTCCCCTTGTACAGCTCGTC-3’ |
| mH2C | 239-GCDGFYAC-246 | 5’-GGCAGCCATCATCAC-3’ |
|  | (sfGFP) | 5’-TTAGCACGCATAAAAACCATCGCACCCCTTGTACAGCTCGTC-3’ |
| mH3C | 239-GFHAHP-244 | 5’-GGCAGCCATCATCAC-3’ |
|  | (sfGFP) | 5’-TTAAGGATGCGCATGAAACCCCTTGTACAGCTCGTC-3’ |
| mH4C | 239-GWYAWML-245 | 5’-GGCAGCCATCATCAC-3’ |
|  | (sfGFP) | 5’-TTACAGCATCCAGGCGTACCAGCCCTTGTACAGCTCGTC-3’ |
| mH5C | 239-GWYSWLL-245 | 5’-GGCAGCCATCATCAC-3’ |
|  | (sfGFP) | 5’-TTACAGCAGCCAGCTGTACCAGCCCTTGTACAGCTCGTC-3’ |
| E-mH1 | 135-KCCYSL-137 | 5’-CCGGCGAGTCCGGCGGATCGAGAAGTGGGGCACAAGCTGGAG-3’ |
|  | (EGFP) | 5’-GCCGTCCTCCTTGAAG-3’ |
| E-mH2 | 135-CDGFYAC-137 | 5’-CTTCAAGGAGGACGGCTGCGATGGTTTTTATGCGTGCGGGCACAAGCTGGAG-3’ |
|  | (EGFP) | 5’-CTCCAGCTTGTGCCCGCACGCATAAAAACCATCGCAGCCGTCCTCCTTGAAG-3’ |
| E-mH3 | 135-FHAHP-137 | 5’-AAATGCTGCTATAGTCTGGGGCACAAGCTGGAG-3’ |
|  | (EGFP) | 5’-GCCGTCCTCCTTGAAG-3’ |
| gFPS-HER2-BP1 | 131-KCCYSL-137 | 5’-GGCATCGACTTCAAGTGCTGCTATAGTCTGGGGCACAAGCTGGAGTAC-3’ |
|  | (sfGFP) | 5’-GTACTCCAGCTTGTGCCCCAGACTATAGCAGCACTTGAAGTCGATGCC-3’ |
